# Neuromodulation by mGluRs in Sound Localization Circuits in the Auditory Brainstem

**DOI:** 10.3389/fncir.2020.599600

**Published:** 2020-11-05

**Authors:** Nupur Goel, Kang Peng, Yong Lu

**Affiliations:** Department of Anatomy and Neurobiology, Hearing Research Group, College of Medicine, Northeast Ohio Medical University, Rootstown, OH, United States

**Keywords:** neuromodulation, mGluR, sound localization, ITD, ILD

## Abstract

The ability of humans and animals to localize the source of a sound in a complex acoustic environment facilitates communication and survival. Two cues are used for sound localization at horizontal planes, interaural time and level differences (ITD and ILD), which are analyzed by distinct neural circuits in the brainstem. Here, we review the studies on metabotropic glutamate receptor (mGluR)-mediated neuromodulation of both intrinsic and synaptic properties of brainstem neurons in these circuits. Both mammalian and avian animal models have been used, with each having their advantages that are not present in the other. For the mammalian model, we discuss mGluR neuromodulation in the ILD circuit, with an emphasis on the recent discovery of differential modulation of synaptic transmission of different transmitter release modes. For the avian model, we focus on reviewing mGluR neuromodulation in the ITD pathway, with an emphasis on tonotopic distribution and synaptic plasticity of mGluR modulation in coincidence detector neurons. Future works are proposed to further investigate the functions and mechanisms of mGluRs in the sound localization circuits.

## Introduction

Glutamate, the most ubiquitous excitatory neurotransmitter used in the nervous system, activates two types of receptors, ionotropic and metabotropic receptors (iGluRs and mGluRs). These receptors are tasked with fast and slow neurotransmission respectively. Soon after mGluRs were discovered (Sladeczek et al., 1985; Nicoletti et al., 1986a,b), studies on their structure, functional expression, and signaling pathways followed (Sugiyama et al., 1987; Houamed et al., 1991; Masu et al., 1991). To date, eight members of mGluRs have been identified. They are classified into three groups based on signaling mechanisms and amino acid sequences (reviewed by Niswender and Conn, 2010). Group I mGluRs consist of two members, mGluR1 and mGluR5. They are typically expressed in postsynaptic cells, and function through G_q_/G_11_ proteins. Group II mGluRs consist of mGluR2 and mGluR3, and group III mGluRs have four members (mGluR4, 6, 7, and 8). Both group II and group III mGluRs are expressed primarily on presynaptic loci and function through G_i_/G_o_ proteins. Since mGluRs play a crucial role in modulating many neural circuits, mGluRs have been targets for drug development to treat various psychiatric conditions such as anxiety disorders, depression, schizophrenia, and chronic pain (reviewed by Swanson et al., 2005; Krystal et al., 2010; Crupi et al., 2019). This review focuses on mGluR modulation of sound localization circuits in the brainstem. The general topics of mGluRs can be found in other reviews (reviewed by Krystal et al., 2010; Niswender and Conn, 2010; Nicoletti et al., 2011; Tharmalingam et al., 2012; Lodge et al., 2013). Additionally, mGluR modulation of both synaptic transmission and intrinsic excitability of auditory neurons, which is important for modulation of auditory processing, has been reviewed previously (reviewed by Lu, 2014; Tang and Lu, 2018).

Sound localization allows an organism to discern spatial characteristics of sounds, providing a clear evolutionary advantage in terms of communication and survival skills. For sound localization in the horizontal plane, two cues are used, interaural time differences (ITD) and interaural level differences (ILD; reviewed by Grothe et al., 2010). While ITD cues help process low-frequency sounds, ILD cues are well suited for high-frequency sounds (Goupell and Stakhovskaya, 2018). It is conceivable that mGluRs are involved in sound localization pathways, because glutamate is the principal excitatory neurotransmitter expressed from the cochlea to the auditory cortex, and mGluRs are found to be expressed in all these auditory stations (reviewed by Lu, 2014; Tang and Lu, 2018). The brainstem is the preliminary location of integrating complex auditory processes such as sound localization. Understanding the functions of mGluRs in auditory circuits will provide a deeper appreciation for modulatory mechanisms involved with auditory processing and help cultivate potential therapeutic approaches targeting mGluRs in treating hearing disorders such as tinnitus (ringing of the ear without external sound stimuli; reviewed by Galazyuk et al., 2019). Here, we review the studies on mGluRs in the avian ITD and the mammalian ILD circuits with a focus on brainstem structures.

## mGluRs in Mammalian ILD Pathway

**Figure 1:**
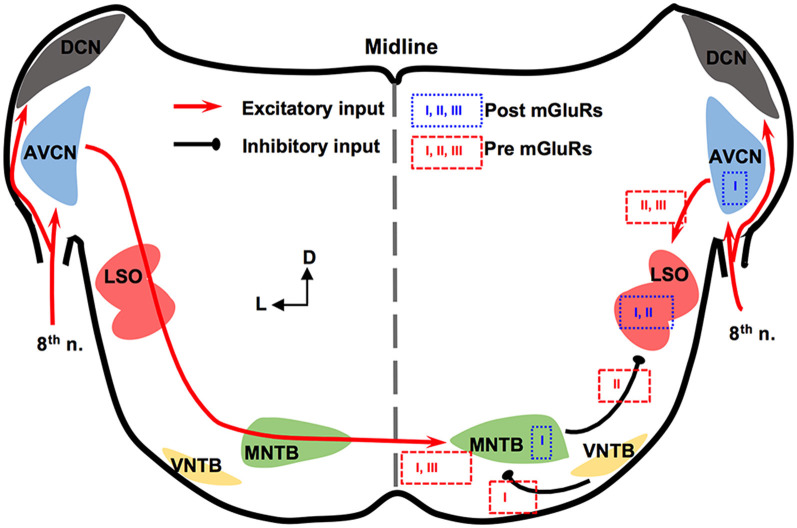
Metabotropic glutamate receptor (mGluR) modulation in the mammalian brainstem interaural level differences (ILD) pathway. Modulation of neuronal properties by mGluRs has been observed at the ILD-coding site lateral superior olivary (LSO), as well as the input nuclei, anteroventral cochlear nucleus (AVCN) and medial nucleus trapezoid body (MNTB). At the LSO, groups I and II mGluRs at the postsynaptic site generate Ca^2+^ signaling. Presynaptic group II mGluRs regulate the inhibitory input from MNTB while groups II and III mGluRs regulate the excitatory input from AVCN. At the AVCN, the group I mGluRs facilitate the excitability of bushy cells. At the MNTB, besides facilitating the excitability, the group I mGluRs exert release-mode dependent modulation on both the excitatory and inhibitory inputs to MNTB. Group III mGluRs may also be involved in modulating the excitatory input to MNTB. The text boxes containing the subtypes of mGluRs indicate postsynaptic (within a particular nucleus) or presynaptic (outside of the nucleus) modulation.

The mammalian ILD pathway is extensive and spans many subcortical and cortical structures. The auditory nerve enters the brainstem and innervates the cochlear nucleus (CN). The CN has three sub-nuclei: dorsal CN, anteroventral CN (AVCN), and posteroventral CN (PVCN). These sub-nuclei have diverse functions and neuromodulation (Farago et al., 2006). Outputs from the AVCN lead to excitation of the ipsilateral lateral superior olivary (LSO) complex. The LSO also receives synaptic inhibition from the ipsilateral medial nucleus trapezoid body (MNTB), which receives excitatory input from the contralateral AVCN and converts the excitation to an inhibitory output. The integration of ipsilateral excitation and contralateral inhibition allows LSO to encode ILDs (reviewed by Tollin, 2003). Thus, our discussion will be focused on mGluRs in AVCN, MNTB, and LSO (Figure 1).

In AVCN, at the mRNA and protein levels, mGluR1 (a subtype of group I mGluRs) is moderately expressed (Shigemoto et al., 1992; Petralia et al., 1997; Bilak and Morest, 1998; Kemmer and Vater, 2001). Chanda and Xu-Friedman (2011) have provided physiological evidence for the presence of tonic activity of group I mGluRs in AVCN. Depolarization of bushy cells by activation of mGluR1 and/or mGluR5 is believed to enhance the excitability of these cells (Chanda and Xu-Friedman, 2011), forming a contrast with the inhibition mediated by GABA_B_ receptors (GABA_B_Rs). For groups II and III mGluRs, very little is known about their expression or function in the AVCN (Ohishi et al., 1998).

The expression and *in vitro* physiology of mGluRs in MNTB has been relatively extensively studied. Group I mGluRs are expressed primarily on the postsynaptic membrane of MNTB neurons (mGluR1: Kushmerick et al., 2004; mGluR5: Peng et al., 2020). Kushmerick et al. (2004) has shown that *via* retrograde signaling, a postsynaptic group I mGluRs (predominantly mGluR1) inhibit glutamatergic transmission at the calyx-MNTB synapse. Remarkably, mGluR5 (and maybe mGluR1 too) is also expressed presynaptically in the calyx of Held, and enhances spontaneous glutamate release, *via* regulating a persistent voltage-gated Na^+^ channel current (Peng et al., 2020). The differential mGluR modulation on spontaneous vs. evoked glutamate release is consistent with our finding that group I mGluRs differentially modulate spontaneously vs. evoked inhibitory postsynaptic currents (sIPSCs and eIPSCs, respectively) based on the neurotransmitter (glycine or GABA; Curry et al., 2018). Group I mGluRs selectively increase glycine sIPSCs while depressing GABA eIPSCs and having minimal effect on glycine eIPSCs and GABA sIPSCs (Curry et al., 2018). Our results (Curry et al., 2018; Peng et al., 2020) support the theory of different synaptic vesicular pools for spontaneous and evoked release and provide potential evidence of multivesicular spontaneous release under mGluR modulation. Besides modulating synaptic properties, the group I mGluRs can also increase the excitability of MNTB neurons *via* different mechanisms from the presynaptic modulation (dos Santos E Alhadas et al., 2019). Activation of these receptors leads to MNTB membrane depolarization, inhibition of inward rectifier K^+^ channels, promotion of action potentials (APs), improvement in the neuron’s ability to follow high-frequency excitatory inputs, and these effects persist into adulthood (P90; dos Santos E Alhadas et al., 2019). The enhancement of cellular excitability of MNTB neurons by the group I mGluRs remains in the presence of the antagonists for the major known ionotropic receptors (Peng et al., 2020), indicating postsynaptic actions of these receptors. Also, K_V_3.1b phosphorylation, which underlies the high-threshold K_V_ conductances, is subject to modulation by the group I mGluRs (Song and Kaczmarek, 2006). Because the high-threshold K_V_ conductances critically define the ability of MNTB neurons to follow spike inputs at high frequency (HF; Johnston et al., 2010), the mGluR modulation of their phosphorylation status affects the inhibitory output of MNTB. Other mGluRs in MNTB are less well understood. Group II mGluRs are shown to be present at synapses of MNTB (Elezgarai et al., 2001). One group III member, mGluR4, is identified at the presynaptic glutamatergic terminals, and its activity is developmentally regulated (functioning before hearing onset; Elezgarai et al., 1999). Functionally, mGluRs make a small (10%) yet physiologically relevant contribution to the presynaptic depression of the excitatory input to MNTB from the calyx (von Gersdorff et al., 1997). Limited data also showed that the excitatory input to MNTB is negatively regulated *via* group III mGluRs (Billups et al., 2005). These studies demonstrate multi-directional regulations by mGluRs of neuronal properties of MNTB. Although MNTB has been considered a simple sign-inverting relay station, the evidence reviewed above suggests rich possibilities for neural plasticity.

In the LSO, mGluR modulation seems to be limited during development. The expression of group II mGluRs is mostly detected in early development (P4) but not after hearing onset (Nishimaki et al., 2007). Supporting this anatomical observation, Ene et al. (2003) showed that the intracellular Ca^2+^ concentration is increased by activation of groups I and II mGluRs in LSO neurons obtained in P0-P4 mice. This mGluR-triggered response in the intracellular Ca^2+^ concentration reduces its amplitude in later animal ages (P20; Ene et al., 2007). Activation of mGluRs (likely groups II and III) inhibits evoked glutamate release at LSO in P14–22 rats, forming feedback control of the excitatory input (Wu and Fu, 1998). Meanwhile, Nishimaki et al. (2007) reported suppression of the inhibitory input to LSO neurons, apparently *via* activation of mGluRs on the inhibitory terminals by glutamate spillover escaped from the synaptic cleft of the excitatory terminals, and the effects diminish a few days after hearing onset. These results suggest a role of mGluRs in the development of the neural circuits involving the LSO.

In summary, mGluRs modulate synaptic inputs to the LSO, and extensively modulate the neuronal properties of the projecting neurons in AVCN and MNTB, constituting a network modulation of the ILD circuit in mammals.

## mGluRs in Avian ITD Pathway

**Figure 2:**
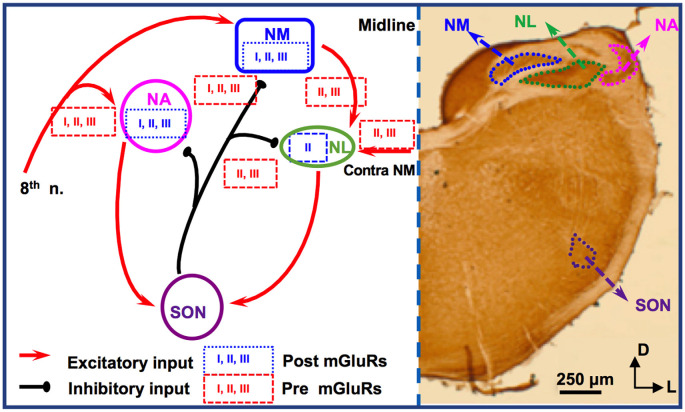
mGluR modulation in the avian brainstem interaural time differences (ITD) pathway. Modulation of neuronal properties by mGluRs has been observed at the ITD-coding site nucleus laminaris (NL), as well as the excitatory input nucleus, nucleus magnocellularis (NM). At the NL, postsynaptic group II mGluRs interact with K_V_ channels, enhancing high frequency (HF) following ability. Presynaptic groups II and III mGluRs co-regulate both the excitatory and inhibitory transmission at NL. Importantly, this modulation varies depending on the coding-frequency region. At the NM, mGluRs on the presynaptic terminals exert fine control on the strength of the inhibitory input from superior olivary nucleus (SON), and postsynaptic mGluRs help maintain Ca^2+^ homeostasis. Data on mGluR modulation of neuronal properties of SON neurons is still lacking.

For studies of mGluRs in the avian ITD circuit, the chicken auditory brainstem represents an excellent model because of its defined and specialized anatomy, and its well-characterized functions (reviewed by Rubel et al., 1990; Grothe, 2003; Burger et al., 2011). There are two subnuclei in the avian CN, the cochlear nucleus angularis (NA) and nucleus magnocellularis (NM), both of which receive excitatory inputs from the auditory nerve (8th nerve). Cells in NM are primarily bushy cells equivalent to bushy cells in mammalian AVCN. The nucleus laminaris (NL), the avian equivalent of MSO in mammals, receives bilateral excitatory inputs from the NM. These two excitatory inputs are morphologically and physiologically symmetrical (Lu et al., 2018). While the NA is tasked with intensity (ILD) cues, NM and NL circuits process temporal (ITD) characteristics. Besides the excitatory inputs, all these three lower brainstem nuclei (NM, NL, and NA) receive synaptic inhibition from projection neurons in the ipsilateral superior olivary nucleus (SON; Burger et al., 2005), as well as from local GABAergic interneurons (Yamada et al., 2013). The SON is driven by excitatory inputs originating from the NA and NL. Therefore, the inhibitory input from the SON onto these lower brainstem nuclei forms a functionally important feedback loop, regulating the synaptic strength of the excitatory inputs to the ITD-coding circuit. The local interneurons are driven by the auditory nerve input and form feedforward inhibition, regulating ITD sensitivity in low-frequency NL neurons (Yamada et al., 2013). Compared to their mammalian counterparts, cells of NM and NL are more homogenous. Our discussion will be focused on mGluRs in NM and NL (Figure 2).

Immunohistochemistry has revealed the expression of group I and II mGluRs in NM neurons (Zirpel and Parks, 2001; Tang et al., 2013). The activity of mGluRs in NM neurons was first reported by Zirpel et al. (1994), in which an increase in the phosphatidylinositol metabolism was observed in response to glutamate. A series of subsequent studies demonstrated the importance of mGluRs in the regulation of Ca^2+^ signaling in NM neurons (Lachica et al., 1995, 1998; Zirpel et al., 1995, 1998; Kato et al., 1996; Zirpel and Rubel, 1996; Kato and Rubel, 1999; Zirpel and Parks, 2001). Because of the high activity of the excitatory input to NM neurons from the auditory nerve, these neurons need to buffer efficiently the activity-induced increase in Ca^2+^ concentration and maintain Ca^2+^ homeostasis for their survival. Modulation of Ca^2+^ signaling by mGluRs constitutes one of the mechanisms, e.g., *via* reduction of Ca^2+^ influx mediated by voltage-gated Ca^2+^ channels in NM neurons (Lachica et al., 1995; Lu and Rubel, 2005). In addition to postsynaptic neuromodulation, multiple mGluRs from all three groups also regulate presynaptic properties of NM neurons, *via* regulation of the inhibitory transmission originating from the ipsilateral SON. The SON inhibitory inputs to NM neurons are unusual because unlike other inhibitory transmissions in the adult brain the GABAergic input to NM elicits depolarizing responses, which exerts an inhibitory action through shunting inhibition (Hyson et al., 1995; Lu and Trussell, 2001; Monsivais and Rubel, 2001). Paradoxically, the GABAergic input could drive the postsynaptic cells to fire APs (Lu and Trussell, 2001; Kuo et al., 2009; Tang et al., 2009), which would be unlikely phase-locked to the excitatory inputs. Such unusual spiking activity could disrupt NM response to excitatory inputs and reduce the phase-locking fidelity of NM neurons, a property essential for accurate ITD coding. Multiple mechanisms have been discovered as a means to regulate inhibitory signals and prevent GABA-induced spiking. One of the important mechanisms, as we review here, is that mGluRs may tonically reduce the inhibitory synaptic strength to NM neurons (Lu, 2007), while a feedback regulation of the GABAergic system is provided by GABA_B_Rs (Monsivais and Rubel, 2001), which may help ensure precise coincidence detection process and ITD coding in the NL.

Synaptic excitation to NL neurons is tonotopically distributed. EPSCs recorded in neurons in HF-coding regions are faster in kinetics and stronger in amplitude compared to low frequency (LF)-coding neurons (Sanchez et al., 2010; Slee et al., 2010). Conversely, synaptic inhibition in NL is also tonotopically distributed. In LF neurons, phasic IPSCs are fast in kinetics and the tonic inhibition is minimal. In contrast, phasic IPSCs are slower and the tonic inhibition is stronger in middle frequency (MF) and HF neurons (Tang et al., 2011; Tang and Lu, 2012a; Yamada et al., 2013). Group II mGluRs are found to be expressed in a graded manner along the frequency axis of NL (Tang et al., 2013). The greatest expression of mGluRs within the NL is in the LF neurons, lesser mGluR expression in the MF neurons, and the smallest expression in the HF neurons. Consistent with the anatomical evidence, physiological results by Okuda et al. (2013) demonstrated that mGluRs regulate EPSCs in NL with different modulation strength depending on the frequency-coding region. In LF neurons, activation of groups II and III mGluRs substantially suppresses the glutamatergic transmission. In contrast, in the MF and HF neurons, the modulation is less strong. This forms a complementary regulation when compared to the modulation by mGluRs of the inhibitory transmission across different frequency regions. Groups II and III mGluRs modulate the inhibitory input to NL neurons (primarily in MF and HF neurons), controlling the synaptic inhibitory strength *via* a mechanism similar to that in NM (Tang et al., 2009). Taken together, mGluR modulation of both the excitatory and inhibitory inputs to NL may help maintain a balance in excitation and inhibition and improve synaptic integration at particular sound frequencies.

Given the symmetrical bilateral excitatory inputs each NL neuron receives from the two ears, the NL serves as an excellent model to address possible plasticity of mGluR modulation. After removing the cochlea from one ear, we witnessed a dramatic reduction of evoked EPSCs and surprisingly a selective increase in group II mGluR expression and physiological suppression of the excitatory input from the deafferented pathway (Lu et al., 2018). The results suggest that unilateral cochlear ablation disrupts the animal’s binaural processing capacity, and the upregulation of mGluRs modulation over eEPSCs presents an interesting case of anti-homeostatic plasticity. It is worth to point out that most of the studies on mGluRs in the avian auditory system have used relatively mature chicken tissues, and the developmental aspects of mGluRs have not been examined except for one study in which the modulatory strength of mGluRs on the inhibitory input to NL gradually increases over age (Tang and Lu, 2012b), in contrast to the mammalian system where mGluRs usually diminish over development. Therefore, the anti-homeostatic plasticity observed after hearing deprivation is unlikely a reversion to the pre-hearing status. Besides, the intrinsic neuronal properties of NL neurons are also regulated by mGluRs in a coding frequency-dependent manner (Hamlet and Lu, 2016). By using a technique that preserves intracellular signaling pathways (perforated patch-clamp recording), we reported that the high threshold K_V_ currents in NL neurons are enhanced, with the strongest modulation in LF neurons. This mGluR enhancement of K_V_ currents renders NL neurons the ability to follow high-frequency inputs, *via* an increase in the membrane outward rectification and sharpening of the waveform of APs (Hamlet and Lu, 2016). We propose that this modulation provides a feedforward modulatory mechanism that enhances temporal processing, especially when the peripheral input is of high intensity. Based on these studies, we conclude that mGluR modulation of neuronal properties in the ITD-coding NL neurons is tonotopically distributed, consistent with the tonotopic distribution of the synaptic excitation and inhibition mediated by their respective ionotropic receptors. The differential modulation by mGluRs of the neuronal properties in different frequency coding regions of the NL represents a great example of fine-tuning of auditory processing within a single nucleus.

## Concluding Remarks and Future Works

Here, we reviewed the studies on mGluRs in the sound localization circuits in both the mammalian and avian auditory brainstem, with an emphasis on the latest discoveries regarding mGluR modulation in the mammalian MNTB and the avian NL. While we have a decent grasp of mGluR expression in these circuits, a huge gap remains in our knowledge about mGluR physiology at the cellular and systems levels. There is no data on mGluRs for ITD-coding neurons in the mammalian MSO and the ILD-coding neurons (in the posterior portion of the dorsal nucleus of the lateral lemniscus). Thus, more studies regarding mGluR modulation of those circuits need to be performed. Research conducted from the 1980s up until 2010s of human brain slices concludes that humans possess an MNTB, MSO, and LSO (Kulesza and Grothe, 2015). These structures play a vital role in auditory processing and provide evidence to believe that ITD and ILD coding are interlinked and multi-dimensional in humans. Thus, our understanding of their proper function in different animal species directly correlates to the understanding we have of their function, or lack thereof, in our species. There are many vital questions about the roles of mGluR in sound localization circuits that still need to be answered. Are the presence and activity of mGluRs essential to the development of the auditory circuits underlying sound localization? Whether and how mGluRs regulate binaural sound processing at the systems level? Can mGluRs be used as potential targets for developing therapeutics to improve binaural auditory processing in cochlear implant patients? These questions warrant more active research in this field and our lab’s ongoing works aim to address some of these unknowns.

## Author Contributions

YL perceived and supervised the project. NG wrote the draft of the main text. KP prepared the figures. NG, KP, and YL edited the article. All authors contributed to the article and approved the submitted version.

## Conflict of Interest

The authors declare that the research was conducted in the absence of any commercial or financial relationships that could be construed as a potential conflict of interest.
